# Socio-Economic Implications of COVID-19 Pandemic in South Asia: Emerging Risks and Growing Challenges

**DOI:** 10.3389/fsoc.2021.629693

**Published:** 2021-02-24

**Authors:** Golam Rasul, Apsara Karki Nepal, Abid Hussain, Amina Maharjan, Surendra Joshi, Anu Lama, Prakriti Gurung, Farid Ahmad, Arabinda Mishra, Eklabya Sharma

**Affiliations:** International Centre for Integrated Mountain Development, Kathmandu, Nepal

**Keywords:** COVID-19, socio-economic impact, public health, Poverty and inequality, South Asia

## Abstract

The dramatic spread of COVID-19 has threatened human lives, disrupted livelihoods, and affected trade, economy and businesses across the globe. The global economy has begun to show major disruptions and is heading toward a severe recession with an unprecedented economic crisis. As the global economy is highly integrated and interdependent through the global supply chains, it has been profoundly affected by the COVID-19 pandemic. Although all countries have faced difficulties due to Covid-19, South Asian countries in particular have had to deal with a more challenging situation due to their large population, weak health facilities, high poverty rates, low socio-economic conditions, poor social protection systems, limited access to water and sanitation, and inadequate living space, necessary to maintain physical distancing and take other required measures to contain this pandemic. To contain the spread of the virus, South Asian countries have imposed stringent lockdowns, which have consequently affected the lives and livelihoods of millions of people in the region, where a third of world’s poor live. Against this backdrop, this paper examines the existing and prospective impacts, risks and challenges of Covid-19 on key social and economic sectors including migration, tourism, informal sector, agriculture and rural livelihoods. The analysis revealed that COVID-19 is likely to affect economic growth, increase fiscal deficit and monetary burden, increase the risks of macroeconomic instability, decrease migration and remittance, reduce income from travel and tourism, and result in dwindling micro-small and medium industries and informal businesses. This is likely to deepen poverty and increase unemployment and the risks of hunger and food insecurity. If not addressed properly, this may reinforce existing inequalities, break social harmony, and increase tension and turbulence. The economic and social costs of the COVID-19 outbreak are therefore likely to be significant and long-lasting in South Asia.

## Introduction

The escalating spread of COVID-19 has posed the gravest threat not only to the world economy but also to lives and livelihoods. What started as a health shock has now been transformed into a global economic crisis. In a heavily globalized and interconnected world, this has translated into a state of unparalleled economic recession (Ozili and Arun 2020). COVID-19 has become a global systemic economic risk as it has affected almost all the economies of the world, no matter how small or large they are. Because of high globalization, economic integration and interconnectedness among the different sectors of economy, a change in any part of the economy or any country now affects other sectors of the economy in other parts of the world as well. Like climate change, pandemics are now global risks as it can spread around the world quickly, regardless of where it originates (Acharya and Porwal, 2020; Ibn-Mohammed et al., 2020).

COVID-19 has posed unique challenges to the South Asian economies due to the region’s large population and high rates of poverty, deplorable health infrastructures, poor socio-economic conditions, inadequate social protection systems, limited access to water and sanitation facilities and inadequate living space arrangements (Rasul, 2020; Hossain, et al., 2020). South Asia is one of the poorest regions in the world: about one-third of the world’s poor live in this region with about 70 per cent living in rural areas and primarily dependent on agriculture. Before the COVID-19 pandemic, 649 million people in South Asia were moderately or severely food insecure and 271 million were severely food insecure. Similarly, 36 percent of the children were stunted and 16 percent were acutely malnourished. The situation is likely to worsen further due to the effect of COVID-19 (Rasul, 2020).

The world’s many megacities such as Delhi, Mumbai, Karachi and Dhaka are in this region and their population density is extremely high. In many countries, people lack access to basic services such as clean water, sanitation and hygiene facilities. For example, close to 42% of households in Afghanistan are compelled to use unsafe drinking water and more than 50% do not have access to water and soap to wash hands (ICIMOD, 2020). Furthermore, high population density, poor working conditions and inadequate living space make social distancing very difficult. The world’s largest slums are in South Asia and many of these are home to huge numbers of people, for example the Orangi area in Karachi, Pakistan (2.5 million), Dharavi in Mumbai, India (1 million), and the Rohingya camps in Cox’s Bazaar, Bangladesh (about one million) (Rasul, 2020). These overcrowded living spaces and limited, and often shared, water and sanitation facilities have made physical distancing and self-isolation difficult, consequently increasing the risks of exposure and vulnerabilities (Hossain, et al., 2020). Because of the already strained economic conditions, the majority of the people in this region have few resources and weak capacity to cope with the exposures of a pandemic shock. The challenges are reinforced by the fact that a large share of population make their living through informal sectors or self-employment, without any health or social protection (ICIMOD, 2020).

Being concerned with the fast spread of COVID-19, a few scholars, particularly in India, have made efforts to understand the nature dynamics of the COVID-19 pandemic to model and forecast the pace of transmission and rates of mortality (Khajanchi and Sarkar, 2020; Samui et al., 2020; Khajanchi et al., 2020). Similarly, Acharya and Porwal (2020) have also assessed whether the population’s vulnerability of being infected and the rates of mortality due to the infection depend on the demographic composition of the population in the different states of India. All these studies emphasized the need for maintaining physical distance and contract tracing to control the spread of the corona virus. Realizing the importance of maintaining physical distance, the governments of this region have imposed strong lockdowns to save people’s lives.

Although South Asian countries have been relatively successful in containing the spread of the virus and saving people’s lives in the early months of the pandemic (both infection and loss of lives are relatively low in South Asia compared to many developed economies), the success has come at a high economic cost due to extended lockdowns which directly impacted economic activities. Simulation results suggest that lockdown of any economy for a month might result in an annual GDP loss of 1.5%–2.0%. It is estimated that the Indian economy incurs a loss of US$ 4.64 billion for locking down the economy for a single day (Acuité Ratings, 2020).

South Asian countries have poor health care systems. Afghanistan has only 2.8 physicians per 10,000 people, Bhutan 3.8, Bangladesh 5.3, and Nepal 6.5, a 10th of the number in more advanced countries. Even India, which has one of the strongest health systems in the region, has only 7.8 physicians per 10,000 people (Rasul, 2020). The South Asian countries, due to weak health facilities and resources, have taken very stringent policy measures to contain the spread of the corona virus and save people’s lives. Except a few essential services, the economic activities have shutdown, travels are banned, movement of goods and services are restricted and cross-border movements are closed. Labor, the main factor of production, has been quarantined, borders have been closed and national, regional and global supply chains have been disrupted mostly in the South Asian region.

While the current policy measures of physical distancing and lockdown are critical for saving people’s lives and in combatting the spread of the corona virus, these measures have affected the lives and livelihoods of millions of people in the South Asian region, which is home to one third of world’s poorest population (ESCAP, 2020). The stronger the lockdown, the greater the economic impacts are. In this backdrop, this paper briefly examines the following questions: What are the existing and prospective economic impacts of COVID-19 pandemic in South Asia, what are the challenges and issues faced by the poor vulnerable population, and what are the likely impacts in the near future—short, medium and long-term? How the economic sectors like, migration, tourism, the informal sector, and agriculture and rural livelihoods will be affected by this pandemic? What are the key measures and actions taken by South Asian countries to address these challenges? What policy responses are needed by national, regional and global communities to address these challenges?

The paper is organized as follows. After this introduction, *Economic Impacts of COVID in South Asia* assesses the macro-economic impacts of COVID in South Asia, particularly on vulnerable economic sectors. *Emerging Social Risks and Vulnerabilities*, examines the cascading effects and emerging social risks and vulnerabilities. *Emerging Opportunities* discusses emerging opportunity to use the disruptive forces of the COVID-19 pandemic and the associated policies for recovery. *Government and Civil Society Responses*, discusses government responses and the final section of the paper draws a conclusion and suggest policy measures that are required to address these challenges.

## Economic Impacts of COVID-19 PANDEMIC in South Asia

South Asian countries took various stringent measures to contain the spread of COVID-19**.** Key measures undertaken by different countries included closure of offices, restaurants, hotels, schools, colleges and education institutions, international borders, suspension of visas, imposition of complete international and domestic travel bans, and ban on public gatherings (Table 1). While these measures significantly helped control the coronavirus spread in South Asia, they also imposed huge economic and social cost at the society. Tourism, exports and remittances, which are important sources of foreign exchange earing for South Asian countries, have also been affected significantly. South Asian economies are likely to shrink for the first time in 4 decades (IMF, 2020). This section summarizes key socio-economic impacts that South Asian countries faces due to the Covid-19 pandemic.

**TABLE 1:** Measures taken by the Governments in South Asia to contain spread of COVID-19 in early stage of infection.

Country	Lockdown measures (duration)	International borders/travel restrictions	Air travel	Land transport	Maritime transport
Afghanistan	Complete lockdown from 22 March till 24 May	Closed, open only for immigrants	Passive restriction enforced by neighbor countries at border	Passive restriction enforced by neighbor countries at border	Not applicable
Bangladesh	Started from 26 March to 16 May and extended further to May 30. Hotspot lockdowns imposed	Closed	Domestic flights opened from 1st June. International flights closed till 15th June. Cargo flights only	Freight trains	Essential good only
Bhutan	Restriction on entry of tourists from 6 March; 23 March—international borders sealed lockdown from April 1 to 21	Closed	Not allowed	Essential goods only	Not applicable
India	25 March to 31 May lockdown extended till 30th June in containment zones. Many activities were allowed after June 8, 2020	Closed	Domestic flights resumed but passive restrictions for international flight	Passive restriction enforced by neighbor countries at border	No data
Maldives	Public health emergency was declared on 19 March. Lockdown from 1 April to 12th June in greater Malé	Closed	Permission required	Not applicable	Allowed
Nepal	23 March to 2 June. Further extended to 14 June	Closed	Permission required	Conflicting information	Not applicable
Pakistan	1 April to 9 May. lockdown lifted	Closed	International flights allowed cargo flights only	Prohibited	Allowed
Sri Lanka	Public holiday declared from 15 March lockdown from 20 March to 11 May	Closed	Cargo flights	Prohibited	No data

Sources; Adapted from UNESCAP, 2020.

### Increasing Risks of Macroeconomic Instability

***Declining GDP growth*:** While the pandemic is still developing and the actual economic impact has yet to be fully known, different forecasts suggest that the South Asian countries will experience the worst economic performance in the last 40 years due to COVID-19. The magnitude of the economic impact will depend upon the duration and severity of the health crisis, the duration of the lockdown, and the manner in which the situation unfolds once the lockdown is lifted. As per the International Monetary Fund’s forecast, the overall GDP growth rate for the South Asian countries is expected to be in the range of -18% (Maldives) to 3.8% (Bangladesh) in 2020, where five countries are expected to have negative growth trajectory (Table 2). A sharp fall of GDP or negative growth of GDP means that a significant part of the population would lose income during 2020. While population is growing and inflation is raising (Table 1), the reduction in GDP means that the per capita income will decline further, which will affect the livelihoods of the general public. If the global economy recovers rapidly, the South Asian region is also expected to have better growth prospect in 2021, where Maldives is expected to lead the region with 12.7% growth followed by India (8%).

**TABLE 2:** Macro-economic indicators of South Asia -Real GDP Growth, Inflation, and Current Account Balance.

	Real GDP growth (annual % change)			Inflation (% change in consumer prices)			Current account balance (% of GDP)		
	Actual	Projections		Actual	Projections		Actual	Projections	
Country	2019	2020	2021	2019	2020	2021	2019	2020	2021
Afghanistan	3.9	−5.0	4.0	2.3	5.4	4.8	11.7	9.5	7.8
Bangladesh	8.2	3.8	4.4	5.5	5.6	5.9	−1.7	−1.5	2.8
Bhutan	3.8	0.6	−0.5	2.6	3.6	4.6	−22.5	−21.4	−13.5
India	4.2	−10.3	8.8	4.8	4.9	3.7	−0.9	−0.3	−0.9
Maldives	5.7	−18.6	12.7	1.3	0.4	2.7	−26.0	−31.8	−17.0
Nepal	7.1	0	2.5	4.6	6.4	6	−7.7	−2.5	−7.0
Pakistan	1.9	−0.4	1.0	6.7	10.7	8.8	−4.9	−1.1	−2.5
Sri Lanka	2.3	−4.6	5.3	4.3	4.7	4.6	−-2.2	−3.6	−3.2

Sources: Internal Monetary Fund, 2020.

***Declining trade volume:*** COVID-19 has severely disrupted international and regional trade and supply chains (Baldwin and Tomiura, 2020). Many countries have temporarily closed their borders, reduced or halted non-essential imports, and canceled import orders from other countries. South Asian countries' growth in the last few decades were fueled by their export growth. The USA, Europe and China are the main trading partners of the South Asian countries, and have themselves been affected badly by the pandemic with their economies slowing down. Because of the sharp drop in external demand, trade and exports contracted sharply as well. COVID-19 has thus heavily impacted both the export and import of South Asian countries. While exports have been growing steadily in recent decades, this year it is expected to be less than that of 2019 in all of the South Asian countries. Overall in South Asia, export growth will be from −6.8 to −3.9% and import growth from −7.3 to −6.2% due to reduced external demand, which will be low even beyond the lockdown period in 2021 (World Bank, 2020a). The reduced export earning is likely to compound the economic crisis in other sectors, like employment and household income. For instance, the textile and garments sector, which employs millions of people and contributes a lion's share of the export earning, is heavily dependent on external markets and will suffer heavily.

***Inflation*** *:* The South Asian countries are expected to experience a slightly higher inflation in 2020 owing to the impact of COVID-19. Except Pakistan (10.7%), all other South Asian countries are expected to have a low level of inflation (0.40% in Maldives to 6.4% in Nepal) in 2020, which is comparable to 2019 inflation rates (Table 1). The current account balance (% of GDP) is expected to be negative for all South Asian countries in 2020, except Afghanistan. The deficit however is expected to be slightly lower than 2019 because of the sharp fall in oil price as well as due to the disruption in global supply chain, where imports are expected to be reduced more than the reduction in exports for most of the countries. South Asia region is a net oil importer and oil occupies the lion's share of the import bill.

***Macroeconomic consequences***. Lower revenue collection and higher recurrent spending are likely to increase the fiscal deficit to 7.7 percent of the GDP in 2020 (World Bank, 2020a). The high fiscal deficits in the region are adding to public debt, affecting fiscal sustainability. Low or negative GDP growth, declining export earnings and increased fiscal deficit have serious implication to household income and poverty. Over time, the macroeconomic crisis will translate to broader macroeconomic challenges that will lead to falling demand and mobility disruptions. The existing macroeconomic crisis may cascade to different economic sectors and compound the impacts at local economy and unfold many economic crizes through both forward and backward linkages and impact both supply (national, regional and global supply chains) and demand (consumption, saving and investment) as well as change prices. If the crisis prolongs, many SMEs may not survive and migrant workers will not be able to return to their original jobs; the recovery could take even longer and these economies may enter into the worst economic recession. The governments of the South Asian countries, therefore, face huge challenges in managing this unprecedented situation, which has major implication for the poor and marginalized communities. With temporary sealing of the border and restriction of the movements, informal cross-border trade is heavily affected, not only putting a number of informal enterprises involved in the supply chain of those goods at a higher risk, but also leading to shortage of groceries, particularly for landlocked countries like Bhutan and Nepal that are heavily dependent on import of basic goods from India.

### Impact on Migration and Remittances

Due to restrictions in travel, mobility and gatherings, the most affected sectors are tourism, sports, entertainment (cinema), education, transport, manufacturing, migration and remittances. South Asian countries rely on foreign remittances as one of the main sources of foreign exchange earnings and household income. With closure of the remittance transfer businesses, loss of employment abroad, and absence of travel back home, remittance inflow in South Asia is expected to decline significantly.

Migration and the resulting remittance is one of the important sources of livelihoods for millions of poor households in the region (KNOMAD, 2020; World Bank, 2020c). The inward remittance from migrant workers serves as a lifeline for their families and as an important source of foreign currency, which contributes significantly to the national economy. For instance, in Nepal, remittance contributes about 27% of the national GDP (2019). India is the largest receiver of international remittance, not only in the region but globally, with US$ 83 billion in 2019. Remittance is also an important source of household income in other South Asian countries where the remittance-GDP ratio was 8.2% in Sri Lanka, 7.9% in Pakistan, 5.8% in Bangladesh and 4.6% in Afghanistan in 2019 (World Bank and KONOMAD, 2020). Like external migration, internal migration also contributes significantly to support families and ensure food and nutrition security for the poorer sections in the rural areas. The rural poor migrate to urban centers and support their families residing in rural areas. India has over 100 million internal migrants, who are also in huge numbers in other countries. The COVID-19 outbreak has placed many internal migrant workers in dire conditions, with many losing their (mostly informal) jobs and unable to return home due to disruptions in public transport services and movement restrictions. This is the reality for most migrant workers, especially those working in the informal sector and living in overcrowded slums. Lockdowns, travel bans, and social distancing measures in response to the COVID-19 crisis have disproportionately affected poor and vulnerable internal migrant workers, who have found themselves stranded, unable to return either to their places of work or their communities of origin. A number of newspaper articles reveal that thousands of workers marched to their villages from cities, despite movement restrictions and lockdowns. Without adequate access to housing, basic water and sanitation, health facilities, or social safety nets to help them survive in such restrictions, these migrant workers have become even more vulnerable to contagion risks. A recent survey in Bihar and UP in India reported that 73% of the respondent migrant households have lost their jobs or main income source (Population Council, India, 2020).

The migration and remittance sectors have been affected heavily by the COVID-19 pandemic. The initial estimates suggest that remittances will fall sharply in all of South Asian countries in 2020. The remittance flow will drop about 23% in comparison to 2019 in India, Pakistan, and Bangladesh. In Nepal is expected to fall by 14%. In total, in **S**outh Asia, remittance flow is likely to decline from US$ 140 billion in 2019 to US$ 135 billion in 2020 and projected to decline to US$ 120 billion in 2021 (World Bank and KNOMAD, 2020). The coronavirus related global economic slowdown, falling oil prices and travel restrictions may also affect the demand for migrant labor and migratory movements, and this is likely to keep remittances subdued even in 2021. These forecasts were done in the initial phase of the lockdown, but recent data suggest that the remittance inflow may not be affected as much when compared to 2019. However, many migrant workers from the region are infected with the virus in their work destinations with some causalities and with millions stranded in need of repatriation, which poses huge challenges and risks.

### Losing Jobs in Informal Sector and MSMEs

**FIGURE 1:**
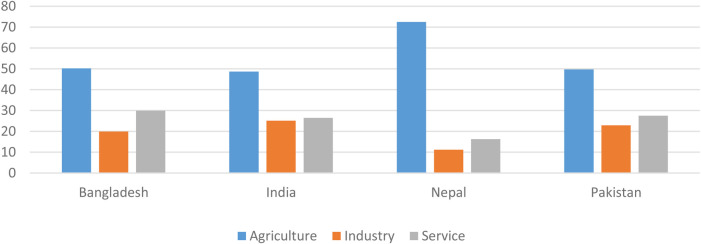
Informal employment in South Asia in agriculture, industry and service sectors. Source: ILO, 2018.

In South Asia, a majority of the population is either self-employed or engaged in agricultural and related activities. Service and industry sectors are other major employers in the region, while micro, small and medium enterprises (MSMEs) and the informal sector services engage the largest workforce. (Figure 1).

For instance, in India 36 million MSMEs employed 60 million people and contributed significantly in national economy (Dev and Sengupta, 2020). In Nepal, MSMEs generates over two million jobs and contributes 22% of the country’s GDP (Shrestha, 2020). In other South Asian countries, MSMEs also play a very important role in providing employment and income, contributing to exports and earning foreign currency.

The informal sector in general and MSMEs in particular have been hit hard by COVID-19 across the South Asia. The informal enterprises and wage labourers face daunting challenges. Many MSMEs are now closed as they could not sustain themselves through the lockdown, leaving many informal sector workers unemployed; economic losses accumulated due to reduced demand, restriction of movement, lack of access to markets, and the loss of mobility of people and goods have all affected workers (ILO, 2020a). COVID-19 restrictions have brought major economic activities to a standstill and have closed the operation of almost all MSMEs except a few health related enterprises. For example, in Nepal, more than a million informal sector workers have lost their jobs temporarily or permanently and are in need of relief materials from the state (Awasthi, 2020). Since hotels and restaurants have been closed for months, demand for food and related materials have also declined sharply.

In Pakistan, about 12 million workers were likely to face layoffs due to lockdown and the country’s sluggish economic recovery (PIDE, 2020). It is also anticipated that if COVID-19 induced situation restricts the GDP growth rate between 0 and 1.5%, it is likely to increase the percentage of poor population (of income poverty) from 25% to around 55% (PIDE, 2020).

The high rate of layoffs and closure of a considerable portion of business will have multiplier effects on employment, household income, food and nutrition security and livelihood security. As most of the workers in the informal sector are poor and the majority of them are women, it has significant implications to poverty, gender and food and nutrition particularly for the marginalized communities who engage heavily on the sector for cash income and livelihood. Food insecurity is another major challenge caused by the disruption of agricultural production, food supply chains, and loss of income across different countries due to the pandemic. At the same time due to the low supply of agricultural production, food prices have been increased leading to severe impacts on household food security. The most vulnerable population in South Asia are those exposed to weather related disasters (flood, droughts), conflict or are living in extreme poverty in countries with weak social protection programs. The strain on incomes resulting from the decline in economic activity will devastate workers close to or below the poverty line and will bring additional people under poverty.

### Inadequate Social Security Coverage

All South Asian countries have lower than world average public health expenditure (Table 3). As percent of the gross domestic product (GDP), the Maldives has the higher share of public health expenditure (5.2%) while Bangladesh has the lowest share (0.47% of GDP). A recent study shows that the some of the cities in South Asia (Karachi in Pakistan and Delhi in India) severely lack intensive care beds, healthcare workers and financial resources to meet the growing demand for healthcare services due to the coronavirus transmission (Davies et al., 2020). Other than Sri Lanka (24%), Bhutan (14%) and India (10%), social security coverage of the workers is quite low where less than 4% of the workers have some kind of social security. Around 140 million workers are expected to lose their jobs in the region (ILO, 2020b; UNESCAP, 2020).

**TABLE 3:** Basic facilities in health sector and Social Security Coverage in South Asian countries.

Countries	Public health exp (% GDP)	Basic sanitation services	Employment with social security	Employment without social security	Potential job losses (millions)
Afghanistan	0.49	38.75	3.7	96.3	12.37
Bangladesh	0.47	43.78	2.5	97.5	
Bhutan	2.49	65.67	14.00	86.00	
India	0.91	50.48	10.3	89.7	112.8
Maldives	5.21	96.2			
Nepal	1.00	51.28	3.4	96.6	2.27
Pakistan	0.72	54.45	3.9	96.1	11.71
Sri Lanka	1.62	93.41	24.1	75.9	0.92
World	5.8	69.94	41.3	58.7	

Sources: ESCAP, 2020.

The following table (Table 4) shows the average score of Global Health Security (GHS) index for South Asian countries. The global average of the GHS score is 40.2. Among the South Asian countries, India and Bhutan are above the global average while the remaining countries (Pakistan, Nepal, Bangladesh, Maldives and Afghanistan) are below the global average score.

**TABLE 4:** Health security ranking and score of South Asian countries.

Countries	Global health security rank and score*	
	Rank	Score
India	57	46.5
Bhutan	85	40.3
Pakistan	105	35.5
Nepal	111	35.1
Bangladesh	113	35.0
Sri Lanka	120	33.9
Maldives	121	33.8
Afghanistan	130	32.3

Source and notes: Higher the score better (lower) the rank. Score is 0–100. 100 is the best health security condition. The rank and scores are based on the following criteria: **A)** Prevention of the emergence of release of pathogens; **B)** Early detection and reporting for epidemic of potential international concern; **C)** Rapid response to and mitigation of the spread of an epidemic; **D)** Sufficient and robust health system to treat the sick and protect health workers; **E)** Commitment to improving national capacity, financing and adherence to norms, and **F)** Overall risk environment and country vulnerability to biological threats. source: Babu et al (2020)

### Effect on Travel and Tourism

Some of the most affected sectors in South Asia due to COVID-19 are tourism, hotel and restaurant, manufacturing, construction and real state, agriculture, transport, trade and so forth (ESCAP, 2020). In the region, travel and tourism sector created around 50 million jobs in 2018 (Table 3), contributing significantly to the national GDPs. For instance in India, tourism and travel services employ about 43 million people contributing over 9% of the GDP; in Pakistan, it contributes over 7% of the GDP; and in Nepal, it employs over one million people contributing about 8% of the GDP (Table 5).

**TABLE 5:** Employment and economic contribution of tourism and travel industry in South Asia.

Country	Share of GDP (%)	Number of jobs in T&T (in Thousand)	Share in total employment, most recent year (%)	Growth (2018 or latest year)
Bangladesh	4.4	2,414	3.9	11.6
India	9.2	42,673	8.1	6.7
Maldives	66.4	69	32.4	7.9
Nepal	7.9	1,051	6.7	3.6
Pakistan	7.1	3,850	6.3	7.4
Sir Lanka	12.5	1,000	12.1	12.4

Source: World Bank (2020a).

The COVID-19 mitigation measures including social distancing and travel restrictions have affected the travel and tourism sector the most. Demands in the tourism, travel, hotel and restaurant sectors collapsed immediately after the spread of the coronavirus, which consequently affected the travel and tourism industry severely. For instance, on March 12, the Nepal government canceled all Everest expeditions slated for 2020 spring season. In the previous years, the Department of Tourism used to collect approximately US$ 4 million annually in royalties from Everest climbing permits. In addition to permits, each climbing team used to spend US$ 40,000–90,000 for other expenses (ICIMOD, 2020). The tourism sector, which generates huge revenue and provide employment to a considerable portion of people, has therefore been extremely affected (ICIMOD, 2020).

It is estimated that in the trekking sector alone, thousands of people will be severely affected while approximately 20,000 tour guides will lose their jobs (De Silva, 2020). The hospitality sector, which employs up to 60,000 workers in Kathmandu, Pokhara and Chitwan, has already been severely impacted by the drop in tourism (Shrestha, 2020). Similarly in India, 40–50 million job cuts are imminent from big hotels, travel agencies and tour operators (Dev and Sengupta, 2020). Bhutan incurred a loss of US$ 4.4 million and Bangladesh lost US$ 470 million from both domestic and international tourism (UNWTO, 2020). Because of the travel ban, the airlines industry is the hardest hit and unlikely to recover soon.

The COVID-19 pandemic is expected to affect the tourism demand and supply in South Asia differentially. At the demand side, international inbound tourists are expected to be reduced, while regional and domestic tourists to be increased. From the supply perspective, loss of jobs and closure of businesses run by MSMEs in the short term, are expected to recover and revive from the medium term onwards due to a growth in regional and domestic tourism in the region.

### Impact on Agriculture and Rural Livelihoods

Due to the COVID-19 pandemic, agricultural value chains and livelihoods of the agriculture dependent population has been suffering (Morton 2020; Sulser and Dunston, 2020). In South Asia, majority of the rural population depend on agriculture and agri-related activities. Agriculture in this region is labor intensive and employs over 50% of the respective countries' labor force. Because of its high labor intensity, agriculture based rural economy and livelihoods are disrupted by COVID-19 and resultant quarantine, restrictions on movement of goods and services and closure of cross-border trade (Sulser and Dunston, 2020; Rasul, 2021). The COVID-19 induced disruption affects the agriculture and the entire food system – the production, transportation, marketing, distribution and consumption. Out of panic, even a few grain exporting countries in the early months of covid-19 restrictions have restricted their exports, which disrupted the international trade, eroded confidence on the global food market and cultivated insecurity (IFPRI, 2020).

COVID-19 has disrupted agricultural operation in the South Asian region because of shortages of labor and inputs, as shut-downs extended to rural areas, village roads, transportation and marketing of goods, all to control the movement of people in order to effectively curb the spread of the pandemic. The outbreak was initially experienced during the planting and harvesting season of many crops, including wheat and paddy, the two major staple foods in the region (Rasul, 2020).

Most affected sub-sectors are fruits, vegetables, poultry and dairy. For example, the poultry sector in Nepal has been losing Rs 220 million per day in recent months (Shrestha, 2020). Fruits are the major cash crops in the region, and the sector was affected badly due to transportation ban and lack of storage and processing facilities. Similarly, dairy farmers could not sell milk; beekeepers could not migrate their bees for spring blooms or get buckets for collection/storage of harvested honey and perform honeybee colony multiplication work/queen rearing, and people could not collect/harvest non-timber forest products (NTFPs) either.

### Shocks in Agriculture and Food Security due to the COVID-19

Although in developed countries the impact of COVID-19 on agriculture is relatively less, in South Asia the impact is substantial because of less mechanization and high labor intensity in agriculture. Across South Asia, rural populations depend on agriculture and agriculture-related activities for their livelihoods; about 50% of the workers are engaged in agriculture (Rasul, 2020 ; Rasul, 2021). While the current policy measures of social distancing and lockdown are critical to save people’s lives and for combating the spread of the corona virus, these measures have affected the agricultural operations with many migrant workers unable to participate in agricultural activities. The disruptions arising from COVID-19 responses have impacted agricultural activities and its supply chain, including the marketing, transportation, distribution and consumption of agricultural goods and inputs in South Asia (Rasul, 2021).

The intensity of COVID-19 shocks on agriculture in the South Asian countries is high because the timing of the COVID-19 pandemic outbreak coincided with the planting and harvesting season of many crops including wheat, paddy, fruits and vegetables. For instance, in Bangladesh, farmers could not deliver harvested watermelon to markets due to transportation bans (Das et al., 2020; ICIMOD, 2020; van Bodegom, and Koopmanschap, 2020). According to FAO (2020), the pandemic restrictions in Bangladesh severely hampered the country’s export of tropical fruits. Additionally, due to travel restrictions, seasonal labourers could not reach the agricultural sites for the Boro rice harvest, which accounts for over half the nation’s rice production. Marketing and selling of poultry, dairy and fruits have also been affected severely in many South Asian countries, particularly the hill and mountain regions. Despite government many efforts, urban poor households in Bangladesh faced acute food insecurity during the lockdown period (Das et al., 2020).

COVID-19 has disrupted food transportation and supply chain in different parts of South Asia. Because of transport restriction and market disruptions, prices of farm products have collapsed and farmers have had to sell their harvested products at very low rates. For instance, farm prices for wheat in India have declined substantially due to lack of facilities to transport the harvest to the markets (Dev and Sengupta, 2020). Similarly, demand for poultry has also shrunken considerably. While prices of farm products have declined, the consumer price of many essential food items increased in almost all the South Asian countries during the initial outbreak of the COVID-19. During the lockdowns most of the countries experienced higher prices of food items and even shortage of food. The situation was further aggravated by the restriction of cross-border movement of goods and trade. A few of the major grain-exporting countries also restricted their exports out of fear of domestic food shortages, which disrupted international trade and regional food markets, and caused acute scarcity in import dependent countries. Border restrictions furthermore affected the transport of agricultural inputs such as chemical fertilizers, seeds, and farm equipment. A decline in food and inputs trade affected food availability in remote areas and may have caused price hike in food importing countries like Afghanistan, Bhutan, Maldives and Nepal (ICIMOD, 2020). For instance, in Afghanistan, initially food prices increased by 30% in Kabul when its border with Pakistan closed, with wheat flour prices increasing by 80–100% in March (Rahim, 2020). Meeting food and nutritional requirements in many South Asian households has been a challenge due to the increasing food prices and loss of jobs during the period of the lockdown. Many poor households have been pushed to cut their expenditure on food items which has compromised their nutrition. Several households have even been forced to borrow money or use their savings to buy food. As estimated by the United Nations University, the COVID-19 pandemic will push 16 million people in South Asia into extreme poverty (Sumner et al., 2020; UNESCAP, 2020). Moreover, a global economic slowdown is highly likely to force international migrant workers to return to their home countries, thus drying up vital foreign exchange resources in many South Asian countries which will consequently affect their food purchasing power.

### Socio-Cultural Impacts

Physical isolation caused by the lockdowns has impacted social relationships, social interactions, and shed light on deep-rooted social norms and exclusions all over the world including South Asia. Sudden layoffs and loss of work have led to depression, alcoholism, substance abuse, and in some cases suicides (Hossain et al., 2020). Although poor and disadvantaged groups suffers more. Newspaper reports and videos circulating on social media record thousands of migrant workers stuck at national and international borders, unable to return to their own homes expressing a sense of abandonment, unfair treatment, and rage. The negative psychological impacts of the pandemic and measures to contain it are raising concerns about mental wellbeing, especially that of senior citizens, frontline healthcare providers, and individuals with existing health problems. The restricted mobility caused by lockdowns has been especially challenging for chronic patients and the differently abled who require regular medical care, but in many cases, have been unable to access it. There is very little public information available regarding the conditions of those living in state institutions—prisons, mental health institutions, shelter homes, and orphanages (ICIMOD, 2020). Many students whose campuses are closed find themselves stranded, often very far from their homes and many express a sense of hopelessness. Sudden layoffs and loss of work have led to depression, alcoholism, substance abuse, and in some cases, suicides. Closure of schools has also denied to children of poorer households, access to mid-day meals which could have adverse effects on nutrition, resulting in increased rates of stunting (UNESCAP, 2020). It is also reported domestic abuse of women has sharply increased during the lockdown period in many parts of South Asia (ICIMOD, 2020). The covid-19 pandemic has intensified the existing inequalities and further creating new forms of exclusion.

## Emerging Social Risks and Vulnerabilities

Social protection system is very limited across the South Asian countries, where vulnerable population is expected to face unprecedented challenge due to the COVID-19 pandemic at their own expense. A huge share of populations in the region is involved in informal work with daily wage labor. The COVID-19 pandemic is likely to bring additional risks and challenges for South Asia, impacting communities and households through multiple channels. The poorest of the poor households and communities have already been impacted through the collapse/reduction of tourism, sharp fall in migration and remittances (both external and internal), and loss of jobs in the urban areas. Informal jobs in the cities and urban areas have evidently shrunk. Large numbers of internal migrant workers have returned back home and many international migrants are also returning or will have to return to their respective countries of nationality in the short- or medium-term. These initial impacts of COVID-19 will, however, permeate to other sectors including agriculture.

Besides the loss of income from job and migration, the South Asian population will suffer from the dampened demand of their products and services due to fall in aggregate demand and avoidance behavior (e.g., tourism, travel, recreation) due to ongoing risks of contracting the virus. Moreover, the loss of income and fall in remittances may increase indebtedness and force households to sell their productive assets and/or severely restrict the ability of the poor households to invest in education of their children, and/or compromise on their nutrition, which will have long-lasting effects. This may deepen poverty, inequalities and vulnerabilities in the region. The economic and social costs of the COVID-19 outbreak are likely to be significant and long-lasting.

Besides few online schooling, most of the schools are closed and millions of children are out of school. It is expected that many children, especially girls, marginalized communities, and the disabled may not be back to school, as they will be forced to make up for their household income loss caused by the pandemic. Table 6 presents the existing drivers, emerging risks and challenges, and potential implications for South Asia.

**TABLE 6:** Emerging social risks and challenges and possible socio-economic implications.

Drivers/triggers	Emerging risks and challenges for south Asian countries	Socio-economic implications
∙Corona virus hits in waves and there is huge uncertainty about the future of the spread of the corona virus ∙Corona virus continues to spread and the economic slowdown continues ∙Extended lockdown	∙Increased uncertainty about future of the spread of the corona virus ∙High risk of fiscal deficit and macroeconomic instability ∙Devastating economic impacts especially for mountain economies ∙Increased lockdown and devastating impacts on the regional economy	∙Higher risks and vulnerabilities in mountain areas ∙Increased health risks and inaccessibility to health services ∙Overburdened health systems ∙Prolonged economic recovery and high economic and social costs
∙Shrinking global demand, reduced exports, increased spending on COVID-19 risk management and social protection	∙Increased risks on macroeconomic stability and investment for economic recovery ∙Increased fiscal deficit and monetary burden due to declining revenues and increased public expenditure	∙Compounded economic challenges ∙Slow economic recovery ∙Increased pressure on already fragile livelihoods
∙Declining informal employment when factories, construction sites, hospitality/service sector and other informal businesses are closed in the cities ∙Slow revival of MSMEs ∙Some SMEs do not survive the crisis ∙Reverse migration takes place in mountain areas	∙Increased unemployment and underemployment ∙Dwindling MSMEs and informal businesses ∙Declining household income ∙Increased economic vulnerability ∙Losing development gain and increasing poverty	∙Double burden for mountain economies and increased economic vulnerability ∙Losing jobs and income ∙Deteriorated mountain economies and livelihoods ∙Increased need for improving local economic opportunities to engage the returnee migrants in productive activities ∙Increased challenge for creating jobs in mountain areas including in public works
∙Migrant workers cannot return to their original jobs in the country ∙Increased return of migrant workers from abroad due to limited job opportunities in destination countries	∙Decreased domestic and international migration and remittance ∙Increased vulnerabilities of migrant households ∙Increased risk on food insecurity	∙Protracted challenge of repatriation of returnee migrants and engaging them in productive economic activities ∙Increased need for ensuring cross-border migration in the region ∙Undermines economic stability and affects livelihoods ∙Falling into poverty trap
∙Slow revival of tourism due to high avoidance and risk behavior ∙Extended length of avoidance behavior	∙Reduced income from travel and tourism ∙Affected jobs and livelihoods	∙Increased challenge of reviving tourism sector ∙Bhutan, Nepal and other mountain areas may face protracted challenges due to high dependence on international tourists ∙Increased risks and vulnerabilities on mountain livelihoods ∙Loss of livelihoods, indebtedness, loss of productive assets ∙Increased risk of slipping into poverty
∙Reduced household income ∙Decline in people’s purchasing power ∙Breaks in food supply chains ∙Increased uncertainty of agricultural and rural operations ∙Increased restriction on export of food and other important agricultural products	∙Increased risks of hunger and food insecurity ∙Adverse impact on food production and supply chain and on food prices ∙Reinforced inequality	∙Increased indebtedness, loss of productive assets ∙High vulnerabilities and food insecurity ∙Heightened need for maintaining buffer food stocks ∙Increased need for regional cooperation to smooth movement of food ∙Increased need for international cooperation to ensure continued flow of food ∙Long-term adverse impact on future generation
∙Households losing an important source of livelihood and income with high risk of chronic poverty ∙Limited fund availability for poverty alleviation programs ∙Increased cost of living	∙Increased poverty and inequalities ∙Increased strain on social safety nets	∙Exacerbated existing poverty and vulnerabilities ∙Leading to deeper poverty and inequality trap ∙Increased poverty and heightened vulnerabilities ∙Exacerbated existing inequalities
∙Disruption of agricultural production and transportation of foods	∙Increasing food panic buying	∙Undermine mountain food security
∙Soaring unemployment, low income, food insecurity and increasing level of anxiety among people may generate discontent and frustration fueling violence and conflict	∙Increased social tension, disturbances and crimes ∙Increased gender and social conflict	∙Exacerbated existing inequalities and social tension and conflicts ∙Chronic poverty sliding back in the hills and mountains ∙Deeper poverty and inequality traps ∙Reinforced inequality ∙Increased social tension, disturbances and crimes

It is evident from Table 4 that the Covid-19 pandemic has led to multiple risks and challenges. Due to the restriction on travel, tourism, and gathering of people to maintain physical distancing, the demand for all kinds of goods and services are suppressed due to forward and backward linkages. As a result, trade volumes (both imports and exports) have declined considerably. However, the domestic spending on health sector, and social security has been increased, but tax revenue declined which have macroeconomic implications.

As a large share of economic activities in South Asia are informal, the closure of factories and restaurants, and the decline of tourism and demand for goods and services, has shrunk the region’s labor absorption capacity. As a result, unemployment rates have soared while incomes have declined (CMIE, 2020). This has made millions of households vulnerable to poverty.

When factories, construction sites, travel and tourism sectors closed, migrant workers returned back to their villages. This return-migration trend is also taking place at the international level, where migrant workers working in foreign countries have lost their jobs and are returning back home. Returnee migrants need additional support for their survival and in the absence of such support system, their families are more likely to be vulnerable to poverty since households not only need to support additional members but have also lost remittances. Covid-19 is likely to deepen poverty and reinforce inequality, increase social tension, disturbances and crimes in South Asia.

## Emerging Opportunities

The current situation provides a unique opportunity to use the disruptive forces of the COVID-19 pandemic and the associated policies for recovery; to accelerate the transition to more sustainable and resilient societies (Rasul, 2020). Some of the short-term measures to address the challenges of COVID-19 can be linked to economic growth by investing in natural capital to improve the long-term productivity and resilience in the region. This requires strategic thinking and strategies for long-term investment to ensure that short-term actions result in long-term benefits. Short-term support can be linked to long-term socio-economic growth through appropriate planning and strategizing which will improve the social and environmental conditions for the sustainable recovery of the health and economic sectors (Rasul, 2020). For example, food for work programs can be linked to programs that construct or maintain local infrastructure such as roads, irrigation canals, management of watershed thus helping poor households to cope with vulnerability while building assets that are essential for society. Similarly, requirements to include energy efficiency in building designs can be linked to support provided to building construction companies to restore jobs, thus providing job restoration in the short-term and climate benefits in the long-term.

### Leveraging Civil Society and Private Innovation

The lockdown has also prompted actors involved in supply chains to adapt a number of important private sector innovations to cope with the pandemic restrictions (Sulser and Dunston, 2020). E-commerce has been growing steadily in the recent years in South Asia due to increased digital connectedness and the development of information and communications technologies. During this period, for instance, consumer-led groups on Twitter, Facebook, and WhatsApp have organized with Farmer Producer Organizations in several countries to find ways of bringing food to markets (Narayanan and Saha, 2020). Many farmers began delivering produce directly using WhatsApp to secure aggregated orders in housing cooperatives in nearby cities (Narayanan, 2020). In India, Swiggy, a fast-growing food delivery app and logistics company, delivers for 40,000 restaurant partners, helping them with its “jumpstart package” to recover sales, while the Swiggy Capital Assist Program helps pay for hygiene and distancing upgrades. During the farmers’ markets shut down, some farmers traveled to cities to set up shop at roadsides maintaining physical distance. In India, Flipkart is growing fast during the COVID-19 crisis and developed a “hyperlocal delivery”grocery service linking SME suppliers with domestic supermarket chains like Vishal Mega Mart with its e-commerce operations (The Economic Times, 2020). Nepal’s nascent e-commerce sector has also growing steadily since the lockdown started.

Global energy demand has declined sharply due to the mitigation measures of COVID-19 and energy price is also going down sharply. Since most of the South Asian countries are net energy importers, the falling oil price could benefit these countries. The reduced oil prices will not only lower the import bill but also help save foreign exchange and will have positive impacts on the current account balance. For example, India is the fourth largest consumer of oil in the global market and a rough estimate suggests that “a US$ 10 fall in crude could reduce the current account deficit by approximately 0.5% of GDP and the fiscal deficit by around 0.1% of GDP” (Sandeep Nayak, The Economic Times, January 12, 2015). The lower oil prices will have positive impacts on manufacturing, cost of fertilizer production for agriculture, costs of transportation and many other energy dependent sectors. The Pakistan government has already declared a reduction in oil prices by Rs. 20 per liter (DAWN, 2020). The decrease in oil prices will decrease production cost and can have positive impacts on managing inflation and living expenses.

Another sector with future growth potential is health services and medical goods and services. Because of COVID-19 pandemic, people are now more conscious about health and the governments may thus invest more in strengthening health facilities. This is likely to increase the demand for health and medical products, including food items with health benefits. This could create an increased demand for health and medicinal products, including the foods with nutritional benefits. The pandemic and resultant disruption in supply chain has created a need for proper development of local economy, local food system, and both on- and off-farm activities. Other important areas of emerging opportunities could be the development of internet-based service sectors. The pandemic has created additional demand for internet-based economic activities, such as online shopping, distance education, as well as online medical services and work from home, which may change the demand for office space and travel needs. Policies that reduce job market frictions and facilitate labor adaptation to these job opportunities would be needed, once the pandemic is controlled in working toward a self-reliant economy for the region.

The COVID 19 pandemic also provides an opportunity to build resilience in the most vulnerable region through the use of stimulus packages to tackle the poverty and other issues. The government may plan strategically to prevent risks and improve resiliency, for instance by promoting sustainable development to reduce the impact of other shocks, such as natural disasters, in the future. As South Asian countries have committed to meet the SDGs by 2030, the governments should utilize their resources and invest in assisting the people affected by the COVID-19.

## Government and Civil Society Responses

The governments of South Asian countries have responded promptly and adopted several policy measures to contain the spread of the COVID-19, support the poor people to ensure food, and provide stimulus to economic sectors. In this section, we briefly present the economic response measures taken by South Asian countries.

### Response to Support Vulnerable People

The major focus of government response on preparations to contain the spread of the virus, increase resources for the health-care system including financial support for medical testing and treatment of the disease, as well as fiscal support for emergency public interventions to increase hospital capacity and medical supplies. All the governments have strengthened their social protection programmes (cash and asset transfer, including food) and provided resources for supporting poor and vulnerable groups, and provided wage support to low-wage workers. For instance, the Government of Afghanistan allocated US$ 15 million to contain corona virus; the Government of Bangladesh allocated US$ 29 million to fund the COVID-19 preparedness and response; the Government of India allocated US$ 22.6 billion to provide essential food items, health facilities, fuel and direct cash to support the poor people and senior citizens; and the Government of Pakistan introduced a relief package worth US$ 7 billion for next three months. The governments of Bhutan, and Nepal also adopted different social protection measures to support poor and vulnerable groups. For example, Nepal government took full responsibility of bearing the cost of testing and treatments of people who have been infected by the coronavirus. In some of these countries, monetary incentives and support was also provided to encourage people to comply with the quarantine efforts. Besides government support, NGOs, civil societies, private sector and religious organizations also extended their support in providing food and essential items to the poor. In Pakistan, government has Ehsaas program to strengthen coordination with NGOs and other civil bodies to target the poor population for delivery of rations more effectively.

### Response to Minimize the Short-Term Economic Pain

Besides supporting vulnerable people, governments of South Asian countries have also adopted various fiscal and monetary measures to minimize the short-term economic pain and to inject liquidity into the financial system. The governments have adopted different measures including concessional lending to prioritized sectors, reduced restrictions on payments such as forbearance of taxes, rent and utility payments and deadlines for loan payments to make liquidity available and to provide flexibility for debtors. For instance, the Reserve Bank of India rolled out a plan of 1.8% of GDP to increase liquidity in the financial sectors. In addition to the central government efforts, some States in India also adopted stimulus measures to sustain economic activities and support the poor. For instance Kerala State has announced US$ 2.6 billion package (2.5% of the state GDP) for economic recovery and some direct transfers to poor households (World Bank, 2020a). Similarly, Government of Pakistan introduced a huge stimulus package and financial support including US$ 600 million support to SMEs and tax refunds to the export industry (World Bank, 2020a). Government of Bangladesh announced the plan of US$ 588 million salary support to garment and other export industries to support their workers. Government of Nepal also introduced concessional loans for SMEs to pay their workers.

### Response to Reopen and Revitalize Economy

Different countries adopted different strategies for gradual opening of the economies and providing financial support to priority sectors to revive economic activities. Government of India has adopted a measure of zoning the country based on the prevalence of the coronavirus cases to relax the lockdown and gradually restart economic activities in areas where the virus is absent or low in number, yet taking stringent measures in the hotspot areas. The state government of Assam, India has constituted a task force to design strategies to revive the state's economy. India’s central bank has introduced measures to increase liquidity to increase access to credit for the pharmaceutical, construction and tourism industries. Bangladesh government is providing 50–70% subsidy to farmers for buying harvesters to address the challenges of labor shortage in paddy harvesting. The governments of Bhutan, Myanmar and Nepal have also rolled out different measures to stimulate SMEs (World Bank, 2020a). It is heartening that Benapole- Petrapole land border between Bangladesh and India has opened recently after three weeks of lockdown to facilitate movement of people on emergency ground (ICIMOD, 2020).

## Conclusion and Policy Recommendations

The COVID-19 pandemic has posed a huge risk and severely impacted the socio-economic condition and livelihood of people in South Asia. The coronavirus is still spreading and it is difficult to predict when it will be completely contained. The unprecedented challenge posed by the COVID-19 pandemic calls for very urgent and decisive actions to ensure that people’s lives are saved, livelihoods are protected and the economy recovers. The Covid-19 outbreak has caused direct high costs on human health and economic activities, and poses the most adverse effects on livelihoods of the poor and the most vulnerable communities. This study discusses some of the crucial key points that may help assist vulnerable group of people who are suffering from this pandemic. Since the coverage of social security system is minimal or absent altogether in most of the South Asian countries, the government should manage to give some sort of social security facilities to the poorest population, more specifically when they lose their informal employment opportunities. Improving saving habits of the poor and providing access to banking services would, for instance, provide safety nets during times of crisis.

As health, environment and social issues are interconnected, concerted efforts are required to mitigate and recover from the damages brought by the COVID-19 on our societies and economies. Governments need to prioritize their activities for short, medium, and long-term. Regional and global cooperation is also necessary to address the ripple effects of COVID-19 on different societies. The countries of South Asia must act collectively to address their challenges and to create favourable conditions for economic recovery. Importantly, innovative strategies and approaches are needed to address the coronavirus challenges. The governments of the South Asian countries may consider the following policy measures to mitigate the negative impacts of the pandemic on the poor and most vulnerable sections of the society and to promote economic recovery.

1 **Plan for an economic recovery from COVID-19** to develop a strategy to adapt quickly based on the situation, avoiding blanket lockdowns so that areas with low intensity risk are not affected due to closure of the local economies, since blanket shutdown of the economy has taken a devastating toll on the economy and people’s livelihoods. The Indian approach of categorization of the country based on the prevalence of the corona virus cases and opening the economic activities in areas where virus is absent or low, and introducing stringent measures in hotspot areas could be a good starting point. Detailed guideline, however, is necessary to operationalize such policies, such as following clear protocol developed based on local condition and evidence as well as clear strategies for containing the virus after opening. Due to the lack of such protocols, the risk of resurgence of the COVID-19 virus is quite high in these countries, as it is has evidently been occurring in the United States and European countries. It is important to use the media intensively to raise awareness among public and disseminate targeted health care education and self-protection as well as supporting mental health (Khajanchi et al., 2020).
2 **Develop roadmap for achieving short, medium, and long-term goals** to revitalize the national and sub-national economy by taking into account the specific condition and needs of the poor and vulnerable groups at sub-national level. In the short term, focus should be on addressing the immediate health crisis, ensuring food and nutritional security, shorter-term job creation and transferring incomes to the needy population to survive the economy. Medium term focus should be on boosting economic activities to recover the economy and in designing and implementing the best possible stimulus to achieve financial recovery. The long-term goal should be **t**ransforming or bouncing the economy forward by promoting long-term sustainable growth and poverty reduction.
3 O**rchestrate the fiscal, monetary and development interventio**n in an integrated and coordinated manners so that different policy measures complement each other and multiply their effects in economic recovery. While the short-term focus will be on addressing the impacts of the pandemic and restoring jobs and employment, the long-term focus could be on improving long-term productivity and resilience by investing in a balanced portfolio of physical, human, social and natural capitals, which will build capacity to deal with future challenges and mitigate the impact of future pandemics and other socio-economic shocks. For example, investment in health, education, skills development, innovation, technological upgrading, and green infrastructure and natural capital will increase the productive capacity of the population and provide sustainable returns for future generations.
4 **Redouble the efforts of social protection** to protect the poor, vulnerable groups, and migrant workers and compensate the loss of income so that they can maintain minimum standard of living and do not slide back to poverty. Investment in social protection and job creation will be needed to protect the vulnerable in the short term, but policy priorities could gradually shift to reducing the environmental risks affecting human health and vulnerability to climate change. Protecting and enhancing natural capital such as forests, soils, water resources, ecosystems, biodiversity, air quality, and climate can support human health and productivity and improve long-term resilience. For example, investment in green infrastructure such as renewable energy can supply clean energy and improve air quality, which leads to long-term health benefits and positive climate outcomes
5 ***Boosting economic*** *activity and i*nvesting in job creation in areas where poor and lowly skilled workers can participate and get benefits. In striving for sustainability, policy choices, and investment decisions should be arranged strategically in such a way that they not only address immediate problems but also build long-term resilience,** strengthen the existing poverty alleviation program** and targeted poverty reduction programs in asset building and economic recovery.
6 **Promote regional cooperation** to facilitate expedient cross-border movement of essential goods and services including medicines, medical equipment, foods, and other essential items. South Asian countries must work together to address the challenge of pandemic and fast and sustainable economic recovery.
7 **Review and revise the policy priorities** by withdrawing subsidies from fossil fuels and providing subsidies to green recovery, improving health facilities, boosting economic activities, and focusing on programs that alleviate poverty and vulnerabilities. The drop in oil prices offers an opportunity to withdraw the existing subsidies on oil and other fossil fuels and invest more on renewable energy sectors that support green jobs. Local food system should also be strengthened at community levels to improve local food self-sufficiency and nutritional outcomes.

## Data Availability

The original contributions presented in the study are included in the article/Supplementary Material, further inquiries can be directed to the corresponding author.
